# Virucidal Potential of 3,3′,4,4′,5,5′-Hexahydroxy-*trans*-Stilbene Against Respiratory Syncytial Virus

**DOI:** 10.3390/v17101287

**Published:** 2025-09-23

**Authors:** Zoltán Bánki, Leonie Wolf, Brigitte Müllauer, Daniel Geisler-Moroder, Wegene Borena, Walter Jäger, Thomas Szekeres

**Affiliations:** 1 Institute of Virology, Medical University of Innsbruck, 6020 Innsbruck, Austria; leonie.wolf@i-med.ac.at (L.W.); brigitte.muellauer@i-med.ac.at (B.M.); daniel.geisler-moroder@i-med.ac.at (D.G.-M.); wegene.borena@i-med.ac.at (W.B.); 2 Department of Pharmaceutical Sciences, Division of Pharmaceutical Chemistry, University of Vienna, 1090 Vienna, Austria; walter.jaeger@univie.ac.at; 3 Department of Laboratory Medicine, Medical University of Vienna, 1090 Vienna, Austria; thomas.szekeres@meduniwien.ac.at

**Keywords:** respiratory syncytial virus, antivirals, polyhydroxyphenol

## Abstract

Respiratory syncytial virus (RSV) infections have a significant impact on global health. Despite of recent advancements, the current treatment options for managing severe RSV infections remain primarily limited to supportive care, emphasizing the high priority for the development of effective antiviral therapies. Antiviral activity of 3,3′,4,4′,5,5′-hexahydroxy-trans-stilbene (HHS), a synthetic polyhydroxyphenol, has previously been demonstrated against SARS-CoV-2. In this study, we provide evidence for a direct virucidal effect of HHS against RSV infection in permissive HEp-2 cells in vitro. HHS, with an IC50 of 3.44 μM, efficiently inhibited HEp-2 cell infection with no sign of toxicity at least up to 25 μM. Interestingly, resveratrol, a polyhydroxyphenol analogue, was less efficient. Mode of action experiments revealed that HHS directly interacts with RSV particles, indicating that its virucidal activity is based on this interaction rather than affecting HEp-2 cells or post-infection processes. Together with previous data, our results suggest a broad antiviral activity of HHS against different respiratory viruses. Further studies are necessary to unveil the exact mechanism and evaluate the potential of HHS in the treatment of severe respiratory virus infections.

## 1. Introduction

Respiratory syncytial virus (RSV) represents a pathogen that usually causes mild, cold-like symptoms but poses a significant global health burden, particularly among infants, young children, and the elderly. While most individuals recover within one to two weeks, those in the vulnerable groups mentioned before are more likely to develop serious lower respiratory tract infections. According to one of the most recent global estimates from 2019, RSV was responsible for approximately 33 million cases of acute lower respiratory infections among children under the age of five, leading to around 3.6 million hospital admissions, 26,300 in-hospital deaths and 101,400 RSV-attributable overall deaths [1]. Several RSV vaccines have recently been licensed, including the prefusion F protein–based vaccines Arexvy (GSK) and Abrysvo (Pfizer) for adults ≥ 60 years, as well as maternal immunization with Abrysvo to protect young infants [2]. In addition, long-acting monoclonal antibodies, palivizumab (Synagis, AstraZeneca) and nirsevimab (Beyfortus, Sanofi/AstraZeneca), have been approved for passive immunization in infants [3,4]. While these are major advances, important limitations remain: vaccine use is currently restricted to specific target populations, long-term durability of protection in the elderly is uncertain, and immunocompromised patients were largely excluded from pivotal trials. Thus, RSV continues to cause severe lower respiratory tract infections in infants, the elderly, and patients with underlying disease, and seasonal outbreaks place a major burden on healthcare systems. Despite these alarming statistics and the latest advancements, the current treatment options for managing severe RSV infections primarily focus on supportive care, rather than curative approaches [5]. Developing antiviral therapies that can directly target RSV would potentially reduce the severity and duration of the disease, decrease hospitalisations, and prevent complications.

Botanical antimicrobial substances are intensively studied for their potential use against infectious diseases [6]. Resveratrol (RES; 3,4′,5-trihydroxy-*trans*-stilbene), a stilbenoid compound, is produced in various plants including grape vines, pines, berries, and legumes in response to pathogens, UV-radiation and ozone exposure [7]. RES is a small polyphenolic molecule that, in addition to its natural antimicrobial role in plants, has demonstrated potent antimicrobial activities [8]. The antiviral activity of RES has been documented against various enveloped viruses, including herpes simplex virus (HSV), varicella-zoster virus (VZV), human cytomegalovirus (HCMV), Epstein–Barr virus (EBV), human immunodeficiency virus (HIV), influenza A virus, and SARS-CoV-2 [9,10,11,12,13,14,15]. 3,3′,4,4′,5,5′-hexahydroxy-*trans*-stilbene (HHS) is a synthetic small molecule that exhibits enhanced biological effects compared to RES and other related analogues. It has been shown to inhibit cyclooxygenase-2 and ribonucleotide reductase, and demonstrates potent anti-tumor effects not only in various tumor cell lines but also in a murine melanoma model [16,17,18].

Here, we investigated the antiviral activity of a synthetic polyhydroxyphenol, HHS, as well as its naturally occurring analogue, RES, against RSV infection.

## 2. Materials and Methods

### 2.1. Chemicals

RES and its synthetic analogue HHS were generated using standard chemical methodologies as described previously [16].

### 2.2. Virus Stock

For the propagation of RSV and the RSV infection assays, HEp-2 cells were cultivated in DMEM (Sigma Aldrich), with the addition of 2 mM L-glutamine (Thermo Fisher Scientific, Vienna, Austria) and 2% FCS (Thermo Fisher Scientific, Vienna, Austria). The experiments were conducted using the RSV Long strain (kindly provided by T. Grunwald, Frauenhofer IZI, Leipzig, Germany). Virus stocks were generated through the infection of HEp-2 cells, as previously described [19]. Briefly, 20 × 10^6^ HEp-2 cells were incubated with 0.1 MOI (2 × 10^6^ infectious units (IU)) of RSV in 2.5 mL DMEM without supplement for 3 h. After incubation, 50 mL DMEM supplemented with 2 mM L-glutamine and 2% FCS was added, and 10 mL of infected HEp-2 cells (4 × 10^6^) were seeded in 75 cm^2^ culture flasks. After three days of cultivation, supernatants were collected, centrifuged for 10 min at 1000× *g* at 4 °C, aliquoted and stored at −80 °C until use. Virus titers were determined on HEp-2 cells by infection with serially diluted virus stocks. The number of RSV-infected cells was determined after 24 h by using 20 µg/mL of the RSV-specific recombinant antibody palivizumab (Synagis, AstraZeneca, Vienna, Austria), followed by incubation with 4 µg/mL Alexa Fluor™ Plus 488-conjugated polyclonal goat anti-human IgG (Thermo Fisher Scientific, Vienna, Austria). RSV-infected cells were analysed by the detection of green fluorescent foci using an ImmunoSpot^®^ analyser (C.T.L. Europe, Rutesheim, Germany).

### 2.3. RSV Infection Assay

HEp-2 cells were infected with RSV as described previously [19]. Briefly, a predetermined dilution of RSV (corresponding to an MOI of 0.01) was utilised to yield approximately 300 infected cells 24 h post-infection. RSV infection was conducted either in the absence (RSV only) or presence of varying concentrations (0.048–100 µM) of HHS or RES. With regard to the vehicle control, dimethyl sulfoxide (DMSO) was added to the cells at the same concentrations used for HHS and RES. Following a 24 h period of infection, infected cells were determined as described previously [19]. Briefly, RSV-infected cells were probed using 20 µg/mL of the RSV-specific recombinant antibody palivizumab followed by incubation with 4 µg/mL Alexa Fluor™ Plus 488-conjugated polyclonal goat anti-human IgG. RSV-infected cells were analysed by the detection of green fluorescent foci using an ImmunoSpot^®^ analyser. Percentage of inhibition was determined relative to the infection levels observed in untreated RSV-infected HEp-2 cells, which were defined as 100% of the infection levels.

To assess the mechanism of RSV infection inhibition of HHS, HEp-2 cells were infected with RSV in the presence of 8 µM HHS. In a prophylactic setting, HEp-2 cells were pre-treated with 8 µM HHS for 2 h at 37 °C prior to exposure to RSV. Subsequent to pre-treatment, cells were either subjected to a washing procedure in order to remove residual HHS, or were left unwashed prior to infection. Furthermore, RSV was pre-incubated with 8 µM HHS for a period of 2 h at 37 °C prior to application for infection of HEp-2 cells. As a control for this viral pre-treatment, RSV was also incubated in medium alone for 2 h at 37 °C in order to assess any potential loss of infectivity due to the incubation process itself. In accordance with this condition, potential compound instability was evaluated through incubation of HHS alone (no virus or cells) at 37 °C for a duration of two hours prior to infection. As vehicle control, all conditions were included using DMSO concentrations matching those used for HHS. Finally, to assess the potential therapeutic effect, RSV-infected HEp-2 cells were treated with HHS or DMSO 2 h after infection.

To determine productive infection 7.5 × 10^5^ HEp-2 cells were infected with RSV at an MOI of 0.01 in the absence or presence of HHS (25, 5 and 1 µM) for two hours. Input virus was removed, and cells were further cultivated in 500 µL DMEM supplemented with 2 mM L-glutamine and 2% FCS in 24-well plates. 50 µL supernatants were collected at 0, 24, 48 and 72 h post-infection. Supernatants were processed for nucleic acid extraction (EMAG (Biomeriuex, Marcy-l‘Étoile, France). Following the manufacturer’s protocol, the nucleic acids were eluted into a final volume of 110 µL. Extracted RNA was then subjected to reverse transcription polymerase chain reaction (RT-PCR) for the detection of RSV using the Altona Diagnostics RealStar^®^ RSV RT-PCR Kit 3.0 (Altona Diagnostics, Hamburg, Germany) on the Bio-Rad CFX96 real-time PCR cycler.

### 2.4. MTT Assay

HEp-2 cells were cultured with varying concentrations of HHS or RES (ranging from 0.048 to 100 µM). After 24 h of incubation, cell viability was assessed by adding MTT reagent (AAT Bioquest, Pleasanton, CA, USA). Following a further 2 h incubation, the optical density was measured at 560 nm to evaluate metabolic activity.

### 2.5. Statistics

Differences in RSV infection between RSV-infected non-treated groups, HHS/RES-treated groups, and DMSO controls were analyzed by one-way ANOVA followed by Holm-Šidák multiple comparison tests using GraphPad Prism 10.4.2 (GraphPad Software, Inc., La Jolla, CA, USA). Statistical significance of productive infection in HEp-2 cells was assessed by two-way ANOVA followed by Tukey’s multiple comparison test. *p*-values ≤ 0.05 were considered statistically significant (* *p* ≤ 0.05; ** *p* ≤ 0.01; *** *p* ≤ 0.001; **** *p* < 0.0001). The 50% inhibitory concentration (IC_50_) values of HHS and RES were determined by nonlinear regression analysis ([inhibitor] vs. response—variable slope, four parameters) using GraphPad Prism.

## 3. Results

### 3.1. Antiviral Activity of HHS on RSV Infection of HEp-2 Cells

**Figure 1:**
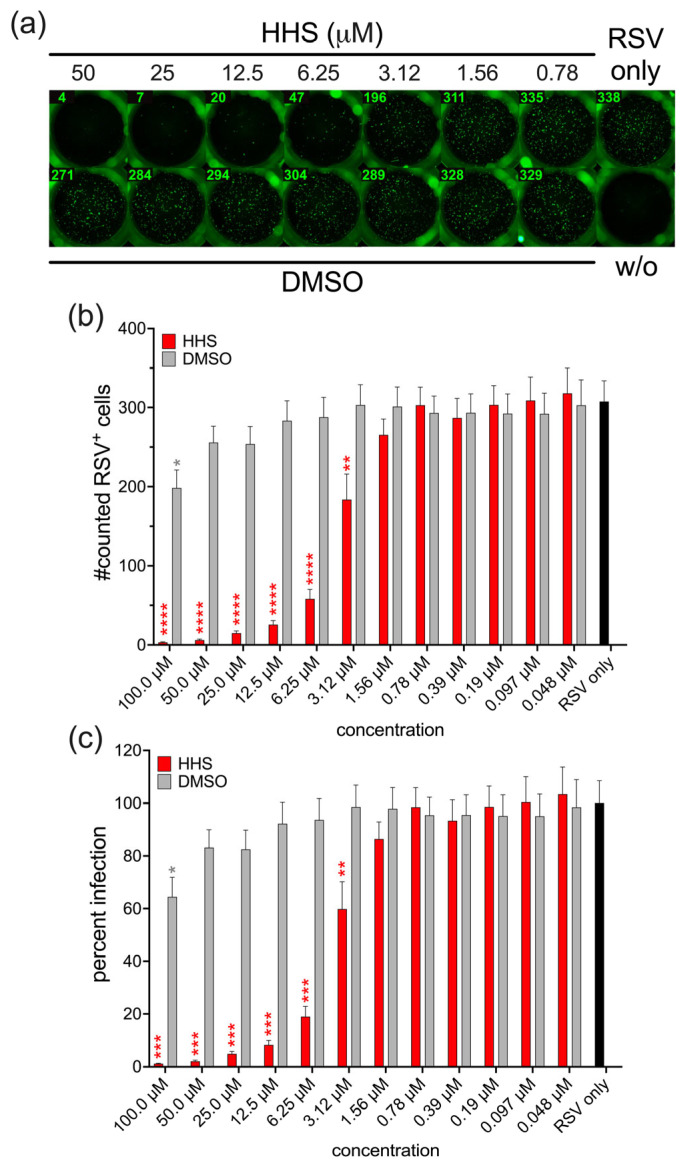
HHS inhibits RSV infection in vitro. HEp-2 cells were infected with RSV in the absence or presence of HHS or the corresponding amount of DMSO. (**a**) A representative result of RSV-infected HEp-2 cells subjected to treatment with HHS, DMSO or uninfected cells (w/o). (**b**) Quantification of RSV-infected cells following treatment with HHS. (**c**) Percent infection relative to the RSV-infected untreated cells (RSV only, set to 100%). Bars represent the mean ± standard error of the mean (SEM) from five independent experiments. Statistical analysis was performed using one-way ANOVA followed by Holm-Šidák multiple comparison tests (* *p* ≤ 0.05; ** *p* ≤ 0.01; *** *p* ≤ 0.001; **** *p* < 0.0001).

To investigate the antiviral activity of HHS, HEp-2 cells were infected with RSV in the presence of HHS at different concentrations. Simultaneously, as a buffer control, RSV infection was carried out in the presence of DMSO corresponding to the amount in the respective HHS dilutions. Infection of HEp-2 cells with RSV in the absence of additives resulted in approximately 300 infected cells per well, as determined by immunofluorescence at 24 h post-infection. The presence of HHS resulted in a dose-dependent inhibition of infection. RSV infection was effectively inhibited by HHS concentrations of 3.12 µM and higher (Figure 1a–c).

**Figure 2:**
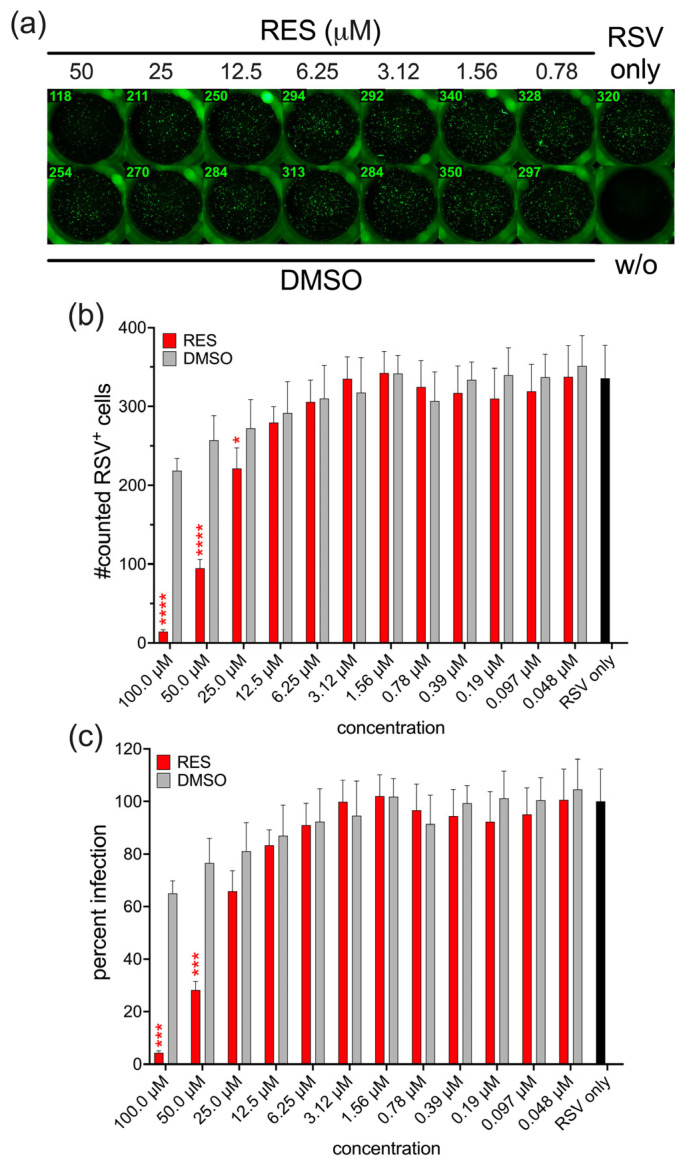
Effect of RES on RSV infection in vitro. HEp-2 cells were infected with RSV in the absence or presence of RES or the corresponding amount of DMSO. (**a**) A representative illustration of untreated (w/o), DMSO-treated, or RES-treated HEp-2 cells upon RSV infection. (**b**) Quantification of RSV-infected cells following treatment with RES. (**c**) Percent infection relative to the RSV-infected untreated cells (RSV only, set to 100%). Bars represent the mean ± standard error of the mean (SEM) from three independent experiments. Statistical analysis was performed using one-way ANOVA followed by Holm-Šidák multiple comparison tests (* *p* ≤ 0.05; *** *p* ≤ 0.001; **** *p* < 0.0001).

Since RES, a polyhydroxyphenol analogue of HHS, has previously been reported to exert antiviral activity in a number of viral infections, we next conducted RSV infection of HEp-2 cells in the presence of RES. Surprisingly, our experiments revealed that the antiviral activity of RES was less efficient compared to HHS. However, RES also showed concentration-dependent inhibition of RSV infections, albeit only at higher concentrations (25 µM and above) compared to HHS (Figure 2a–c).

**Figure 3:**
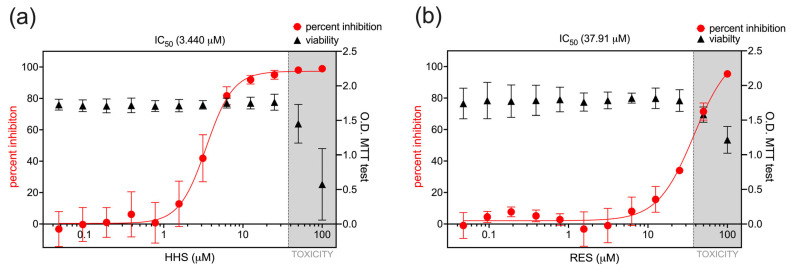
Inhibitory capacity and cytotoxicity of HHS and RES in vitro. HEp-2 cells were infected with RSV and treated with either HHS (**a**) or RES (**b**) to determine the percent inhibition of RSV infection (red dots). The IC_50_ was determined using nonlinear regression, utilising GraphPad Prism. In addition, to assess the potential cytotoxic effects of the agents on HEp-2 cells, the viability of the cells was determined by conducting an MTT test (black triangles). The graphs illustrate the mean with 95% confidence intervals (CIs) derived from five independent experiments for HSS data and three independent experiments for RES.

The difference in antiviral activity between HHS and RES against RSV was more obvious when comparing the IC_50_ values of the two compounds. HHS exhibited an IC_50_ concentration of 3.440 µM, whereas RES required an approximately ten-times higher concentration (37.91 µM) to achieve a comparable inhibitory effect on RSV infection (Figure 3a,b). To confirm that RSV inhibition was not related to potential cytotoxic effects of HHS or RES on HEp-2 cells, we conducted an MTT assay with the cells in the presence of HHS or RES at the concentrations used in RSV infection assays. In the MTT assay with HEp-2 cells, no evidence of cellular toxicity was observed upon application of HHS or RES at concentrations of 25 µM or less. Conversely, higher concentrations of the agents resulted in a decline in the cell viability, thereby indicating the occurrence of toxicity (Figure 3a,b). While HHS achieved near-complete RSV inhibition at the non-cytotoxic concentration of 25 µM (Figure 3a), the IC_50_ concentration of RES falls within the range that could potentially be affected by cytotoxic effects (Figure 3b). Importantly, the 50% cytotoxic concentration (CC_50_) of HHS was calculated to be 54.38 µM, resulting in a selectivity index (SI = CC_50_/IC_50_) of 15.8. An SI of 15.8 demonstrates that HHS exerts virucidal activity at concentrations substantially lower than its cytotoxic threshold, which supports its potential as a candidate for further development.

### 3.2. Antiviral Activity of HHS Related to a Direct Interaction of HHS with RSV

**Figure 4:**
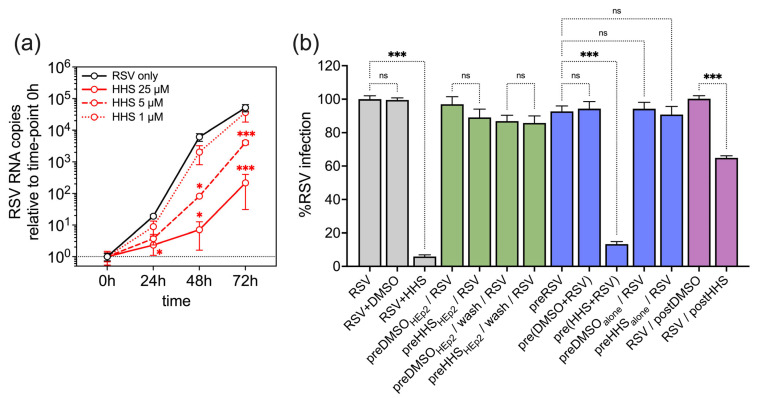
Mode of action of HHS on RSV infection. (**a**) HHS inhibits productive infection of HEp-2 cells. HEp-2 cells were infected with RSV in the absence (RSV only) or presence of HHS (25, 5 and 1 µM) for two hours. Input virus was removed and supernatants were collected at 0, 24, 48 and 72 h post-infection. RSV RNA copies relative to the amount measured at time-point 0 h. Data represent mean values with SD from three biological replicates. Two-way ANOVA followed by Tukey multiple comparison test was used to calculate statistical differences between RSV only and HHS-treated samples (* *p* ≤ 0.05; *** *p* ≤ 0.001). (**b**) HHS exhibits a direct virucidal effect on the RSV particles. HEp-2 cells were infected with RSV and treated with 8 µM of HHS or DMSO under four different treatment conditions. Infection and treatment were either conducted simultaneously (grey bars) or two hours after infection (purple bars). Furthermore, cells were pre-treated (green bars) with DMSO or HHS before infection, with the agent either removed (wash) or left on the cells. Additionally, the agents were pre-incubated at 37 °C alone or with RSV before being added to the cells (blue bars). The data shown is the mean with 95% CI from 4 independent experiments. Statistical analysis was performed using one-way ANOVA followed by Holm-Šidák multiple comparison tests (ns, not significant; *** *p* ≤ 0.001).

Next, we assessed the productive RSV infection of HEp-2 cells with or without HHS treatment. HEp-2 cells were infected with RSV in the absence (RSV only) or presence of HHS (25, 5 and 1 µM) for two hours. Input virus was removed and supernatants were collected at 0, 24, 48 and 72 h post-infection. We found a significant, concentration-dependent reduction in productive infection of HEp-2 cells when cells were infected in the presence of HHS at concentrations of 25 and 5 µM (Figure 4a).

To investigate the mode of action of HHS on RSV infection, infection experiments were conducted using 8 µM HHS (approximately twice the IC_50_ concentration) under four different treatment conditions. In addition to the initial experimental settings, where RSV and HHS (or the DMSO control) were applied simultaneously to HEp-2 cells (Figure 4b, grey bars), HEp-2 cells were pre-treated with HHS or DMSO for 2 h before RSV infection. Following this pre-treatment, HHS was either removed by washing the HEp-2 cells before RSV infection or left on the cells during RSV infection (Figure 4b, green bars).

In contrast to the concurrent administration of RSV and HHS, the prophylactic treatment of HEp-2 cells with HHS did not influence RSV infection, indicating that HHS did not induce an antiviral state of HEp-2 cells or interfere with cell-surface structures necessary for viral entry. Consequently, these mechanisms could be excluded as potential mechanisms for RSV inhibition. In addition, RSV was subjected to pre-incubation with either HHS or DMSO before the addition to the cells (Figure 4b, blue bars). The addition of pre-incubated RSV (2 h alone or with DMSO) to HEp-2 cells did not demonstrate any alteration in the infection of HEp-2 cells. Notably, the 2 h pre-incubation of HHS with RSV resulted in an inhibition of infection on the HEp-2 cells similar to that seen in the simultaneous application, suggesting that HHS directly interacts with RSV itself. The two hours of preincubation of HHS alone before the infection did not show any inhibiting effect on RSV infection, thereby supporting the previous statement and reflecting a relatively rapid loss of HHS activity. As a fourth condition, simulating a therapeutic setting, HEp-2 cells were infected with RSV, and 2 h after infection, HHS or DMSO was added, respectively (Figure 4b, purple bars). The results revealed that HHS also exerts some effect in a therapeutic setting. However, a direct inactivation of RSV attached to but not yet infecting the HEp-2 cells cannot be excluded.

## 4. Discussion

The findings of the present study demonstrate a significant virucidal activity of HHS against RSV infection, which is notably higher in comparison to the efficacy of its natural analogue, RES. HHS exhibited a dose-dependent inhibition of RSV infection, with an IC_50_ value approximately ten times lower than RES, highlighting its superior virucidal potency. These findings align with previous research demonstrating the broad-spectrum antiviral properties of polyphenolic compounds [9,10,11,20,21,22,23] but suggest that structural modifications, such as increased hydroxylation in HHS, may enhance their virucidal effects [24].

The absence of antiviral effects in the prophylactic pre-treatment setting, coupled with the inhibition observed when RSV was directly incubated with HHS, suggests that HHS exerts virucidal activity through a direct interaction with the virus itself, rather than by inducing an antiviral state in host cells. HSS has been shown to inhibit the in vitro proliferation of SARS-CoV-2 even after short-term incubation, suggesting an inhibitory effect on the virus before cell infection [25]. These findings are consistent with previous observations of HHS activity against HIV-1, where inhibition also occurs at an early stage of infection [26]. Furthermore, this mechanism is consistent with previous findings for other polyphenols, which have been shown to disrupt viral envelope integrity or block viral attachment and entry into host cells [27].

Importantly, our data do not provide evidence for inhibition of intracellular stages of the RSV replication cycle independent of direct virus inactivation. As virucidal compounds act primarily by disrupting viral particles and preventing entry, whereas authentic antiviral agents interfere with discrete intracellular steps of the viral life cycle [28,29], we therefore classify the observed effect as virucidal rather than intracellular antiviral activity. Future studies will be required to determine whether HHS may also exert intracellular antiviral effects beyond its demonstrated virucidal activity.

Interestingly, while RES also showed a dose-dependent inhibition of RSV, its significantly higher IC_50_ value and limited efficacy at lower concentrations may reflect differences in chemical structure and solubility. RES has been documented to interact with negatively charged heparan sulfate proteoglycans (HPSG), a putative receptor for RSV on the cell surface, thereby interfering with viral entry rather than directly interacting with RSV’s surface fusion- or glycoproteins [23]. This discrepancy between HHS and RES may be explained by the higher degree of hydroxylation in HHS, which could enhance its binding capacity to viral surface proteins, thereby more effectively interfering with RSV entry or fusion processes [24]. Additionally, in contrast to RES, the complete RSV inhibition achieved with HHS at 25 µM, without observed cytotoxicity, underscores its potential as a promising compound for the development of antiviral drugs.

## 5. Conclusions

Given the critical need for effective therapeutic strategies against RSV, especially in vulnerable populations, our findings suggest that HHS could serve as a valuable candidate for further preclinical development. Future research should focus on in vivo studies to validate the compound’s efficacy and safety in animal models, as well as structural optimisation to refine its pharmacokinetic properties.

## 6. Patents

ZB, WJ and TSz are inventors on a patent application (Application No. EP 24187090.6) related to the findings presented in this manuscript.

## Data Availability

The original contributions presented in this study are included in the article. Further inquiries can be directed to the corresponding author.

## Abbreviations

The following abbreviations are used in this manuscript:

RSV	Respiratory syncytial virus
HHS	3,3′,4,4′,5,5′-hexahydroxy-trans-stilbene
RES	resveratrol
DMSO	dimethyl sulfoxide
